# Impact of control selection strategies on GWAS results: a study of prostate cancer in the UK Biobank

**DOI:** 10.1093/bib/bbag102

**Published:** 2026-03-09

**Authors:** Jingzhan Lu, Johan H Thygesen, Robin N Beaumont, Michael N Weedon, Harry D Green

**Affiliations:** Department of Clinical and Biomedical Sciences, University of Exeter, St Luke's Campus, Heavitree Road, Exeter, Devon, EX2 4TH, United Kingdom; Institute of Health Informatics, University College London, 222 Euston Road, London, NW1 2DA, United Kingdom; Institute of Health Informatics, University College London, 222 Euston Road, London, NW1 2DA, United Kingdom; Department of Clinical and Biomedical Sciences, University of Exeter, St Luke's Campus, Heavitree Road, Exeter, Devon, EX2 4TH, United Kingdom; Department of Clinical and Biomedical Sciences, University of Exeter, St Luke's Campus, Heavitree Road, Exeter, Devon, EX2 4TH, United Kingdom; Department of Clinical and Biomedical Sciences, University of Exeter, St Luke's Campus, Heavitree Road, Exeter, Devon, EX2 4TH, United Kingdom

**Keywords:** GWAS, control selection, matching analysis, biobank, methodology, prostate cancer

## Abstract

As genome-wide association studies (GWAS) studies move from array-based genotyping to whole exome and genome sequencing, there is a significant increase in cost. Applying an appropriate technique for the selection of which controls to include, in large studies where more potential controls are available than needed for the study, may be a useful technique for minimizing resource intensity whilst maintaining statistical power. We evaluated three control selection strategies in prostate cancer GWAS using 15 250 UK Biobank cases: (a) all controls, (b) matched controls, and (c) random selection. Both (b) and (c) achieved comparable power in detecting significant loci relative to (a), but matched controls (b) showed greater consistency in identifying leading single nucleotide polymorphisms (SNPs). However, using (b) matched controls reduced discovery power by ~30% compared with (a) all controls, highlighting a trade-off. Matching controls (1:4 ratio) offers a cost-effective approach for targeted SNP analysis across phenotypes but may miss novel associations.

## Introduction

### Genome-wide association studies

Standard disease genome-wide association studies (GWAS) studies are typically case-control studies aimed to detect genetic variation, known as single nucleotide polymorphisms (SNPs), which associate with the disease of interest. Significant associations between SNP and disease can help understand aetiology and potentially contribute to risk stratification tools [1]. Also, within cancer research, GWAS has been used successfully to identify over 200 genetic risk loci associated with susceptibility to prostate cancer to date [2, 3].

### Control selection further complicated in large longitudinal study

Chatterjee *et al*. emphasized that the validity of case-control studies relies heavily on the appropriate selection of controls [4]. Proper control selection reduces the risk of selection bias and enhances the credibility of study findings. This issue is particularly salient in GWAS using biobank-scale datasets, where vast numbers of potential controls are available, making the strategy for selecting appropriate controls a critical study design consideration.

As digital health infrastructure expands and increasing volumes of electronic health records (EHRs) and biobank-based data become accessible, the challenge of selecting appropriate controls becomes more complex. Prostate cancer is a highly heritable disease, with heritability estimates as high as 58% (95% CI, 51%–63%) according to twin studies [5]. In this study, we will use prostate cancer as an example phenotype because there is a large genetic component to prostate cancer. In the UK Biobank, once prostate cancer cases are defined, virtually any other individual may qualify as a ‘healthy’ control. Therefore, instead of including all eligible controls, we limited our selection to a maximum 1:4 ratio [6], aiming to balance power, reduce bias, and optimize computational efficiency. This study investigates how different strategies for control selection impact GWAS outcomes, with an emphasis on sufficient statistical power and cost–benefit of time and computational resources.

In large-scale cohorts, it is common practice to include all available non-case individuals as controls in GWAS. This issue is especially critical in population-based studies where subtle ancestral differences may remain even after statistical adjustments, leading to both false-positive and false-negative associations [7, 8]. Failure to adequately address this issue remains a key limitation in using large biobank or EHR data where extensive inclusion of all controls without careful matching may compromise the accuracy of GWAS results.

### Cost and scalability challenges in case-control GWAS

Traditionally, GWAS studies have been performed on SNP array data imputed to ~10 million variants, whole genome sequencing (WGS), and whole exome sequencing data (WES) are now more readily available in large cohort datasets such as the UK Biobank [9] and All of Us [10]. Whilst WGS data increases the potential to find novel rare variants, the increased scale of the data causes an increase in computational intensity. Most research in this field is now performed on a cloud-based research analysis platform (RAP) in which researchers pay for the computing resources used, so computational costs also incur a financial cost.

More critically, when conducting new case–control studies, it is not sufficient to sequence only the cases; appropriately matched controls must also be identified and sequenced, which often represents one of the most expensive components of the study. For example, even within large biobank resources, identifying ~3000 prostate cancer cases for whole-genome sequencing also requires sequencing an equivalent or larger number of unaffected male controls. Selecting an appropriate number of individuals and defining a suitable health control cohort can decrease sequencing costs and logistical complexity. Against this backdrop, case-control selection could potentially reduce cost whilst minimizing power loss, especially for studies planning to run multiple GWAS across many phenotypes.

### Matching strategy for control selection

Matching is a commonly employed strategy in case–control studies to ensure comparability between cases and controls by balancing important covariates, thereby minimizing the influence of confounders on case-control study results. Several matching techniques exist, including nearest-neighbour matching, optimal matching, and ratio-based matching. In a comprehensive evaluation using Monte Carlo simulations, Austin compared 12 propensity score matching algorithms, such as optimal matching, greedy nearest-neighbour matching without replacement, and calliper matching, which reported that nearest-neighbour matching consistently demonstrated superior performance. Following these findings, this study, applied a propensity score–based nearest-neighbour matching to select controls with characteristics similar to cases [11].

## Methods

### UK Biobank genotyping quality control and DNAnexus

The UK Biobank (UKBB) genotyping data includes 488 377 participants. All analyses were performed within the secure UKBB Research Analysis Platform (hosted on DNAnexus). The quality control (QC) procedures were based on the publicly released metrics and recommendations provided by the UKBB research team. Samples with a mismatch between self-reported sex and genetically inferred sex were excluded, as were individuals of non-European (non-CEU) ancestry. In addition, 968 outliers based on heterozygosity or missing genotype rate were removed, and samples with sex chromosome aneuploidy were excluded. Variant-level QC excluded genetic variants with an imputation INFO score < 0.7. Minor allele frequencies (MAF) were recalculated within the prostate cancer phenotype subset, and variants with MAF < 1% were excluded.

### UKBB phenotypes and covariates definition

Prostate Cancer cases (N = 15 250) were identified in the UK Biobank using cancer registry records based on the ICD-10 code C61, any participant with this diagnostic code was considered a case. Female participants inferred by genetics were excluded from the analysis. A total of 208 128 male participants without a diagnosis of prostate cancer were available as potential controls. A total of 13 covariates (recruitment age, assessment centre, genotyping chip, and first 10 principal components) were used in the GWAS analysis and matching process.

### GWAS power calculations

A sample size with sufficient statistical power is critical to the success of genetic association studies to detect causal genes of human complex diseases. We performed a power calculation for a case-control GWAS with 15 250 prostate cancer cases and four times the number of controls (1:4 case–control ratio). Power to detect a genome wide significant association for alleles varying in frequency from 0 to 0.03 were tested using an online GWAS power calculator, which used the genpwr R package for power calculation [12]. Using a 1:4 case–control ratio, the power calculation predicted a near-100% power to detect a genome-wide significant association for variants above an allele frequency of 0.03 and an OR of 1.5. This power calculation reflects the theoretical baseline under random control selection, against which the observed gains from random controls can be evaluated.

### Three control selection strategies and GWAS analysis

In this study, we conducted GWAS for prostate cancer within the UKBB using three different control selection strategies to evaluate how the different control definitions affect GWAS results. All controls, included all male participants from the whole population without prostate cancer (N = 208 128); Match controls, selected controls matched to cases based on 13 covariates; and Random controls, included a random selection of male controls. For both strategy match and random, a 1:4 case-to-control ratio was used (N = 61 000). For each strategy, prostate cancer GWAS was performed independently using REGENIE software. We used REGENIE for association testing because it is now considered a standard and up-to-date pipeline for large-scale GWAS, is widely adopted in recent biobank studies, provides high statistical power, and is particularly well suited for UK Biobank–sized datasets [13]. We first ran Step 1 of REGENIE, which used 487 558 variants extracted from UK Biobank genotyping arrays. After linkage disequilibrium pruning and frequency filtering, Step 1 generated a background polygenic model for each participant and chromosome. Step 2 of REGENIE then tested the association of each variant with the phenotype using the predictions from Step 1. The REGENIE parameters were set minor allele count (MAC ≥ 5) and imputed geneotype data based on the TopMed imputation panel [14].

To assess the impact of cdontrol selection on GWAS results, we compared our findings against the largest-to-date trans-ancestry meta-analysis of prostate cancer. This large-scale meta-analysis included 107 247 cases and 127 006 controls across 136 studies contributed [3]. We used the 269 genome-wide significant SNPs reported in Conti *et al*. (2021) as a benchmark, matching them to corresponding SNPs in our GWAS results to evaluate the consistency and effect size of our GWAS results.

### Nearest neighbour matching and random for control selection

To minimize confounding and ensure balance in key covariates between prostate cancer cases and controls, we applied nearest-neighbour propensity score matching using the MatchIt R package (v4.5.2). The nearest-neighbour algorithm was employed without replacement to pair each case with four control individuals having the most similar estimated propensity scores. Matched between cases and controls was assessed using empirical quantile-quantile (eQQ) plots to evaluate covariate balance. Post-matching, matched individuals were retained for downstream GWAS. For Random, we randomly selected four controls for each case from the eligible control population. To ensure reproducibility, a random subset of 61 000 male participants was selected from the full pool of 208 128 eligible controls using a fixed random seed (set.seed = 42).

### Testing the stability of random selection and different case-control ratio

We assessed the stability of the random control selection by repeating this strategy 10 times, using different random seeds (1–10) for each iteration. To reduce computational cost, this evaluation was performed focusing on chromosome 8, which showed the strongest association signal in the main analysis and in Conti *et al.’s* meta analysis. To investigate whether there was a plateau in statistical power when matched case control ratios are increased, we tested integer case control ratios from 1:2 to 1:10 in the matched analysis.

### Locus signal analysis

To identify independent association signals from GWAS summary statistics, we implemented a custom locus-filtering script written in Perl and LocusZoom [15]. The script processes one or more GWAS result files and extracts lead variants based on statistical significance, variant quality, and genomic proximity. For variants passing the filtered criteria, the script then performs locus-level clustering using a distance-based approach. Specifically, variants located within ±500 kb of each other on the same chromosome were grouped as a single locus, reflecting distance-based clumping to account for linkage disequilibrium (LD), a threshold commonly used in genomics [16, 17]. Amongst genome-wide significant variants (*P* < 5 × 10^−8^), a widely accepted threshold in human genetics [18, 19], the variant with the most significant *P*-value (i.e. the highest −log₁₀P) within each locus was selected as the leading SNP. This procedure enables efficient identification of genome-wide significant loci whilst accounting for local linkage disequilibrium and variant quality.

## Results

We characterized the demographic and covariates of the GWAS cohorts to evaluate their comparability (Table 1). The full set of cases (N = 15 250) was contrasted with the three control strategies: all controls (N = 223 378), matched controls (N = 76 250), and random controls (N = 76 250).

**Table 1:** Baseline characteristics of UK biobank participants included in prostate cancer case and control cohorts under different GWAS control selection strategies.

Baseline characteristic	All cases N = 15 250		All-controls^a^ (cases + controls) N = 223 378		Match^a^ (cases + controls) N = 76 250		Random^a^ (cases + controls) N = 76 250	
	*n*	%	*n*	%	*n*	%	*n*	%
Age								
≤ 40	14	0.09%	2555	1.14%	69	0.09%	773	1.01%
40–49	562	3.69%	49 255	22.05%	2934	3.85%	14 855	19.48%
50–59	3514	23.04%	71 261	31.90%	17 278	22.66%	23 241	30.48%
60–69 ≥70	11,001 159	72.14% 1.04%	99 108 1199	44.37% 0.54%	54 972 997	72.09% 1.31%	36 924 457	48.42% 0.60%
Sequencing Chip								
1- BiLEVE array	1710	11.21%	24 946	11.17%	8594	11.27%	8560	11.23%
2- Axiom array	13, 540	88.79%	198 432	88.83%	67 656	88.73%	67 690	88.77%
Recuirment Centre								
11007-Reading	1050	6.89%	13 203	5.91%	4657	6.11%	4531	5.94%
11008-Manchester	857	5.62%	13 223	5.92%	4467	5.86%	4496	5.90%
11009-Newcastle	1000	6.56%	16 517	7.39%	5627	7.38%	5574	7.31%
11010-Leeds	1443	9.46%	19 723	8.83%	6762	8.87%	6746	8.85%
11011-Bristol	1312	8.60%	18 726	8.38%	6250	8.20%	6436	8.44%
11013-Nottinham 11014-Sheffield 11016-Liverpool	1021 922 909	6.70% 6.05% 5.96%	15 188 13 708 14 794	6.80% 6.14% 6.62%	5387 4895 5155	7.06% 6.42% 6.76%	5118 4751 5070	6.71% 6.23% 6.65%

^a^Total Sample size of cases + controls.

We compared the demographic and covariates characteristics of all cases with the three GWAS cohorts (All, Match, Random). Amongst them, the match control selection showed the closest similarity to the case group, especially in age distribution (e.g. 72.1% aged 60–69 in both groups), sequencing centre proportion, and genetic principal components. These results indicate that match control selection method best preserved covariate balance relative to the case group. QQ plots showing distributsions before and after matching can be found in Supplementary Fig. 1. A comparison of the top 10 principal components can be found in Supplementary Fig. 2.

### GWAS power calculation and cost analysis

**Figure 1:**
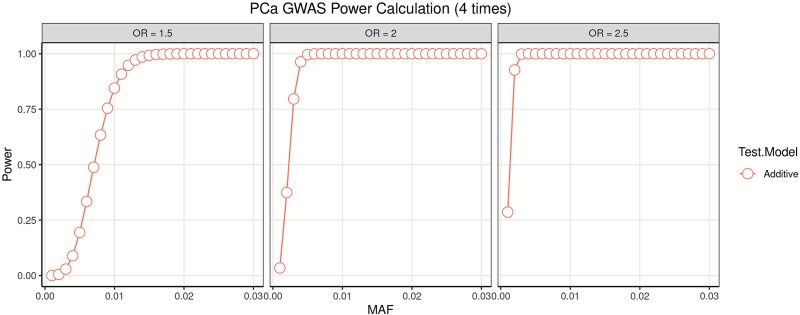
Power estimation for prostate cancer under a 1:4 case-control design. Three panels show additive model power curves for odds ratios (OR) of 1.5, 2, and 2.5. The x-axis represents minor allele frequency (MAF) and the y-axis shows statistical power.

To ensure sufficient statistical power for detecting genetic associations, we performed power calculations in Fig. 1 for a prostate cancer GWAS using 15 250 cases and a 1:4 case-control ratio. Under an additive model, the study reaches over 80% power to detect variants with an odds ratio (OR) of 1.5 when the minor allele frequency (MAF) exceeds 0.007. For variants with ORs of 2 or higher, the study achieves nearly 100% power even at lower MAFs (as low as 0.003). These results demonstrate that a 1:4 case-control ratio using random controls provides sufficient GWAS power and can be generalized to other phenotypes requiring large-scale genetic association studies.

Using a case-control selection strategy reduced the total sample size from 200 000 to 60 000, theoretically offering a 70% reduction in computing resource usage, or 91% reduction for algorithms are of O(N^2) complexity. Sample size reduction and thus resources saved will depend heavily on the prevalence of the phenotype and case/control ratio used. This offers both a cost-saving opportunity and an environmental benefit from reduction in high performance computing usage.

### Manhattan plots of GWAS results

**Figure 2:**
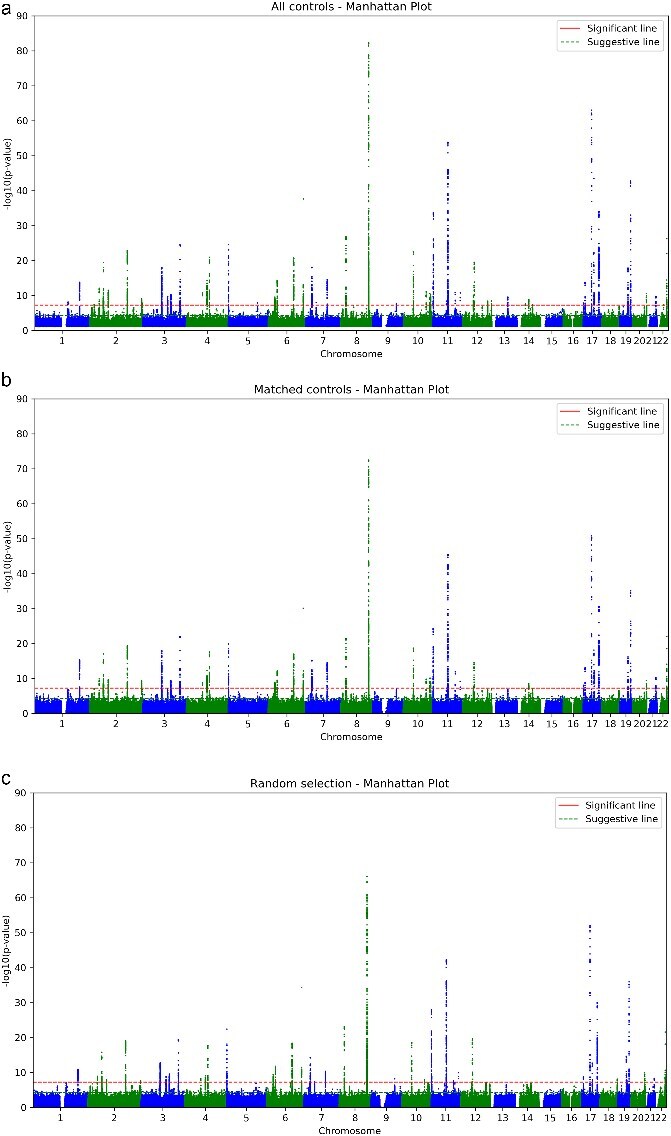
Manhattan plots of GWAS results under different control strategies. Overall signal patterns are consistent, whilst the top –log10(p) peaks differ at 82.3, 72.5, and 66.1.

In Fig. 2, the Manhattan plot revealed genome-wide significant loci peaks are consistent with previously reported prostate cancer susceptibility regions. These consistent patterns further support the validity and robustness of our GWAS findings. However, differences are noted in the upper tail of the *P*-value distribution, where the maximum -log_10_ (P) vary: 82.34 for the All-controls, 72.50 for the Matching, and 66.10 for the Random. Genome coordinates and *P*-values can be found in Supplementary Table S1. A QQ plot showing genomic inflation can be found in Supplementary Fig. 3.

### GWAS significant and suggestive SNPs

We compared the number and concordance of genome-wide significant (−log_10_ (P) > 7.3; *P* < 5 × 10^−8^) and suggestive (−log_10_ (P) > 5; *P* < 1 × 10^−5^) SNPs identified under three strategies: All, Match, and Random (Table 2). The total number of tested SNPs varied slightly across strategies due to control number differences, with All-controls analyzing over 13.5 million SNPs, compared to ~10.9 million in Match and Random.

**Table 2:** GWAS results of significant and suggestive SNPs statistics across three control strategies.

Strategies	Total SNP Numbers	Max –log_10_ (P)	Percentages of SNPs
**All Controls**	**13513780**	**82.34**	
^a^Significant	4572		3.4 × 10^−4^
^a^Suggestive	11 329		8.4 × 10^−4^
**Match Controls**	**10910234**	**72.50**	
Significant	3572		3.3 × 10^−4^
Suggestive	9233		8.5 × 10^−4^
**Random Selection**	**10875211**	**66.10**	
Significant	3064		2.8 × 10^−4^
Suggestive	8503		7.8 × 10^−4^

^a^Significant threshold: *P* < 5 × 10^−8^; Suggestive threshold: *P* < 1 × 10^−5^.

Whilst All and Match yielded comparable capture rates for both significant and suggestive SNPs, Random exhibited a noticeably lower yield in both categories. Specifically, All identified 4572 significant SNPs (3.4 × 10^−4^) % and 11 329 suggestive SNPs (8.4 × 10^−4^) %; Match identified 3572 significant (3.3 × 10^−4^) % and 9233 suggestive (8.5 × 10^−4^) % SNPs; and Random yielded 3064 significant (2.8 × 10^−4^) % and 8503 suggestive (7.8 × 10^−4^) % SNPs. Supplementary Table S2 lists the number of unique loci to each selection strategy. Supplementary Table S3 lists the number of pairwise matches between strategies.

### GWAS locus signal results

The Table 3 displays genome-wide significant association signals (−log₁₀P > genome-wide threshold) detected across three control selection strategies (All, Matched, Random). Shown are loci restricted to chromosomes 8, 10, 11, and 17, which had the most significant findings across the three analyses. Each row represents the lead SNP per locus with –log₁₀P values and allelic information.

**Table 3:** GWAS significant loci with leading SNPs and *P*-values on chromosomes 8, 10, 11, and 17.

**Chr**	**All** **leading SNP**	**Match** **leading SNP**	**Random** **leading SNP**	**All** ***P*-value**^a^	**Match** ***P*-value**^a^	**Random** ***P*-value**^a^
8	23665232 A/G		^b^23668030 T/C	26.92	21.59	23.19
8	127516190 C/G	^b^127518371 G/A	^b^127064901 A/G	82.34	72.5	66.10
10	46098665 C/CA		^b^46099199 C/A	22.67	18.73	18.58
10	103908421 CTT/C			11.09	9.86	7.91
10	121038900 G/A			10.59	10.11	
10	125008517 A/G			10.37	10.13	
11	2211394 C/T	^b^2212255 G/C	^b^2207388 C/T	33.74	24.30	28.04
11	62140968 C/T			8.43	–	–
11	69236512 T/A			53.91	45.54	42.28
11	102530930 C/T			10.93	11.96	7.69
11	125274530 G/A			10.77	9.20	9.99
17	715725 C/T	^b^714799 A/G		7.85	8.11	–
17	7913036 G/T			13.74	13.09	8.78
17	37743574 G/A		^b^37739961 T/A	63.04	50.84	52.13
17	48728343 T/C	^b^48612699 A/AGAGAGC	^b^48654889 T/C	43.55	18.20	12.75
17	71108797 G/A		^b^71120794 G/A	34.09	30.62	30.05

^a^ *P*-value: –log(10) transformed *P*-value here.

^b^Position: The difference leading SNPs located the same loci with all-controls.

The divergence of both Match and Random strategies from the All-controls analysis was notable, with the largest discrepancy observed for random. Amongst the 60 loci identified using All-controls, ~30% leading SNPs were not replicated in the Match group and ˃50% leading SNPs were absent from the Random group. Furthermore, many loci showed attenuated test statistics or inconsistent effect directions in match and especially in random. For instance, lead SNPs at 8q24.21 (chr8:127516190; −log_10_ (P) = 82.34) and 11q13.3 (chr11: 69236512; −log_10_ (P) = 53.91) remained significant in both All and Match, but the corresponding signals weakened or dropped below significance in Random, suggesting reduced sensitivity under random selection. Notably, despite Match and Random detecting a similar number of significant loci, Random often identified different leading SNPs compared to All-controls, indicating frequent lead SNP swapping under random selection.

**Figure 3:**
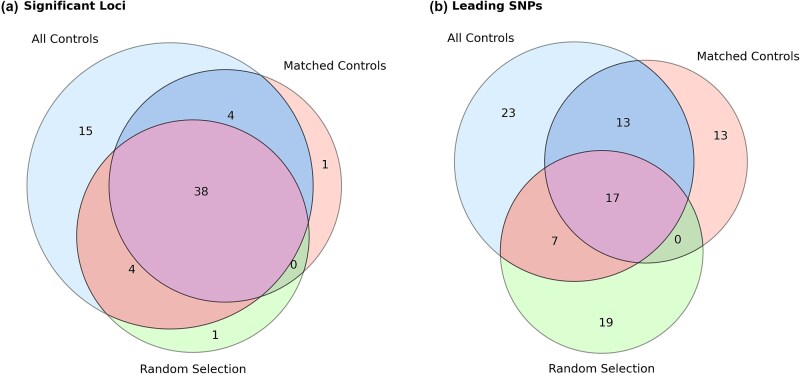
Venn diagram of significant loci and leading SNPs across GWAS from all, match and random cohorts.

A total of 60, 43, and 43 genome-wide significant loci were identified in GWAS using All, matched, and random, respectively. In Fig. 3, shows 38 loci were significant across all three analyses. The overlap between All and Match, as well as between All and Random, was 42 loci in each case, indicating that both matched and random control designs achieved comparable sensitivity in detecting associated regions. However, analysis of leading SNPs revealed greater consistency between All and Match (30 shared SNPs), compared to All and Random (24 shared SNPs), suggesting that the matched design provided more precise localization of association signals. The smaller overlap between Match and Random compared to other pairings (17 shared SNPs and 38 loci, all of which were also shared with All) highlights divergent signal prioritization, implying that random controls may often select alternate tag SNPs within the same loci.

Amongst the unique leading SNPs to the match GWAS (N = 13), 11 (84.62%) were in high linkage disequilibrium (LD; r^2^ > 0.8) with SNPs identified in all GWAS. In contrast, only 11 out of 19 (57.89%) unique leading SNPs from the random GWAS showed such high LD with the all-controls SNPs, indicating improved signal consistency under the matched design. The detailed information of loci and leading SNPs provided in the supplementary table.

### GWAS results show diff strategies OR similar with Conti’s results

**Figure 4:**
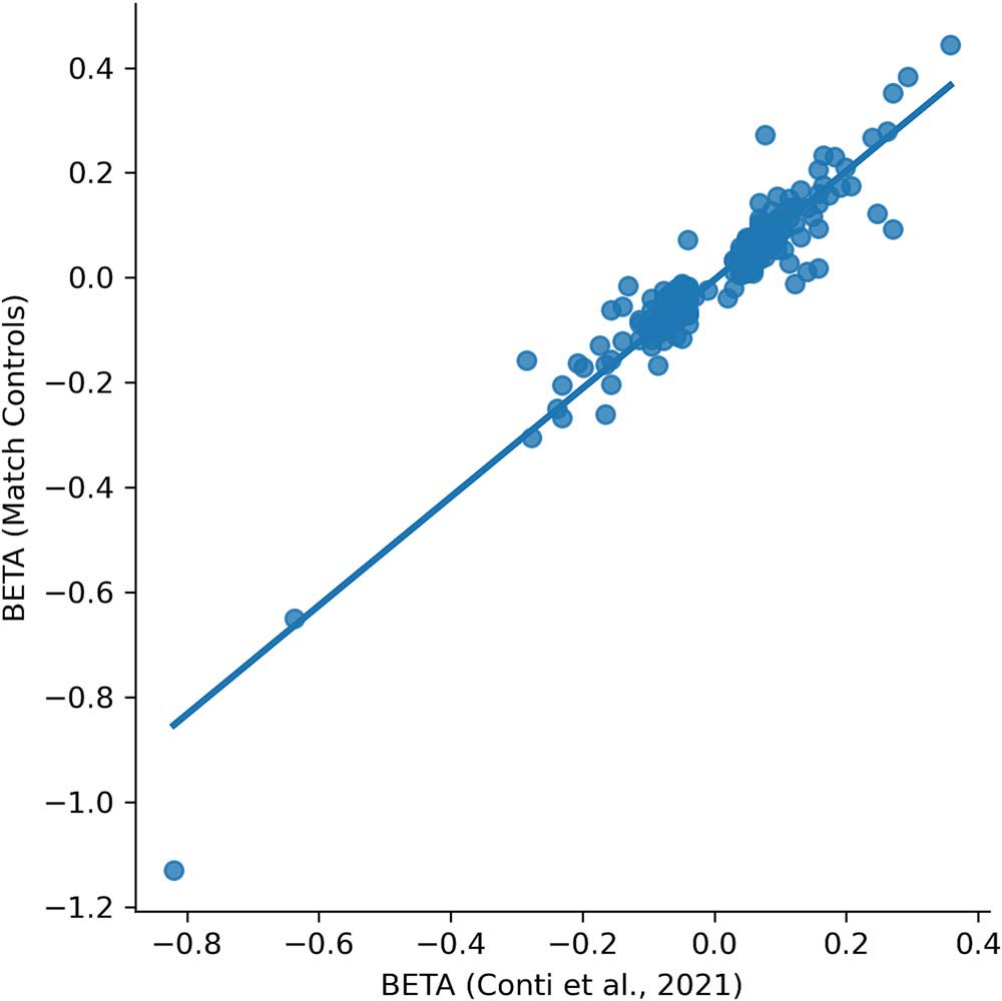
Concordance of 269 SNPs effect size estimates between Conti’s study and GWAS with matched controls.

The scatter plot of Fig. 4 illustrates the correlation between β estimates from Conti *et al*., (2021)’s study (x-axis) and the GWAS results under the “Matched controls” strategy (y-axis). Each point represents a genetic variant (MAF >1%). A linear regression line is overlaid, indicating strong concordance in effect size estimates between the two analysis. Similar linear relationships were observed when using All controls and Random selection strategies. Therefore, only one representative scatter plot (Matched controls versus Conti) is shown. The adjusted R^2^ of 0.82 for the three linear regression models suggests that ~82% of the variance in GWAS effect sizes can be explained by Conti’s results, reflecting high consistency and supporting the notion that ~82% of observed GWAS signals in the real world are reproducible across different control strategies.

### Instabilities of random selection and effects of case–control ratio selection

Across ten repeated GWAS analyses using different random control selections, variability was observed at the locus level. Whilst the original analysis (All controls, match and random on 1:4 ratios) identified two significant loci on chromosome 8, Across the 10 repeated GWAS runs, three rounds (random seed 1, 4, & 5) generated three loci. These additional results were not replicates of a signal found in Conti’s larger-powered study, suggesting false positive results from random variation at low sample sizes. Full results can be found in Supplementary Table S4.

**Figure 5:**
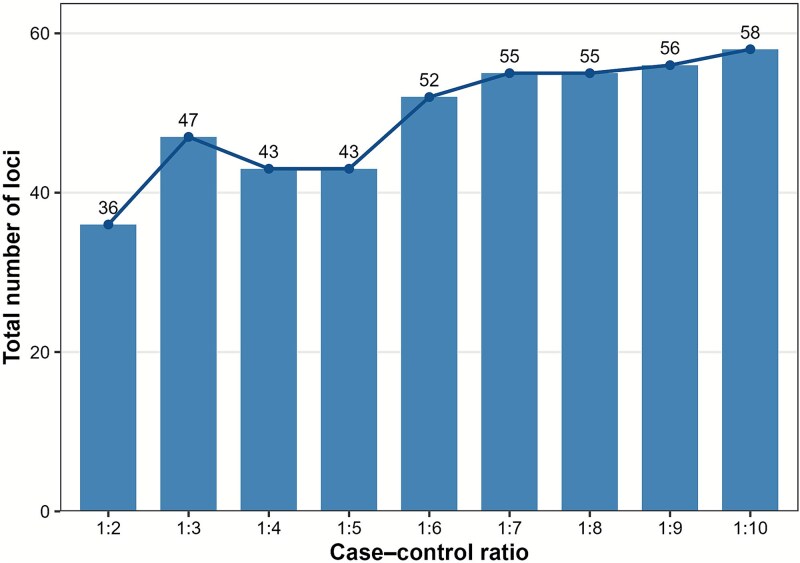
Number of genome-wide significant loci identified in a matched analysis for case–control ratios from 1:2 to 1:10, demonstrating limited increased power past 1:6.

The number of resulting genome wide significant loci as case control ratio increased are in the Fig. 5.The largest gain in loci occurred when expanding the ratio from 1:2 to 1:6, after which the rate of increase became more gradual. Increasing the overall sample size will always increase the statistical power, so we wouldn’t expect the number of loci to stay consistent between our analyses. Instead we aim to determine a balance between the statistical power to discover new loci compared to the cost to perform the analysis. Ratios ~1:4–1:5 appeared to balance locus discovery and noise reduction, which indicate the 1:4 ratio is a reasonable choice to reduce the noise impact. Full results can be found in Supplementary Table S5.

## Discussion

### Principal findings

This study evaluated how control selection strategies influence GWAS outcomes. We compared three distinct approaches: All, Matched, and Random. Whilst all strategies revealed broadly similar global association patterns, reflected in the overall alignment of Manhattan plots and identification of key prostate cancer loci, the depth, and consistency of association signals varied notably across designs.

A similar percentage of genome-wide significant SNPs were identified between all controls 3.4 × 10^−4^ (0.034%) and matched controls 3.3 × 10^−4^ (0.033%), whilst using random controls identified a significantly lower percentage of SNPs 2.8 × 10^−4^ (0.028%). Whilst both Matching and Random selection resulted in a ~30% reduction in genome-wide significant loci compared with All-controls, Matching offered greater consistency with All-controls in terms of leading SNP identification and direction of β estimation.

### Interpretation

Our findings demonstrate empirically the trade-off between computational intensity and statistical power. The increased computational requirements of modern GWAS studies (especially when WGS data is used) incurs an increased computational cost. In our study, the GWAS with All-controls was performed with a total sample size of 200 000 male UK Biobank participants, whilst Random and Matching was performed with 60 000.

Optimal case-control ratios have long been studied in the context of genetic association studies. Whilst increasing the number of controls can enhance statistical power, especially when case numbers are limited, the benefit of adding more controls diminishes beyond a certain point. Empirical and theoretical evaluations suggest that power gains plateau around a 1:4–1:5 case-control ratio, after which additional controls yield minimal improvement relative to cost and computational burden [20,21].

In large-scale studies, controlling for unbalanced case–control ratios and accounting for relatedness amongst samples becomes computationally intensive. Moreover, the risk of population stratification and residual confounding tends to increase with larger sample sizes, as subtle structure in the data can lead to spurious associations [8,22]. This underscores the importance of careful control selection. Matching cases and controls on important covariates such as age, ancestry, and study centre can effectively reduce confounding due to unaccounted population structure and environmentally driven allele frequency differences [23,24].

The utility of case-control matching becomes even more evident in studies of rare diseases. For traits with low prevalence, the ratio of cases to available controls can be extremely imbalanced, often 1:1000 or higher. Whilst using all available controls may seem statistically appealing, such extreme imbalance increases computational burden, especially in models testing rare variants or modest effect sizes [25].

### Strengths and weaknesses

The matching on case-control selection is a plug-in approach to GWAS, which is easy to implement and combine with any pipeline or code module. Demographic comparisons show that matched controls more closely resemble cases in age and genetic background, improving covariate balance, and reducing confounding. This is especially beneficial in large-scale whole genome (WGS) or exome sequencing (WES) studies, where computational and financial costs increase with sample size.

Although both the Match and Random groups are subsets of the overall All-controls group, they represent distinct sampling strategies. Consequently, the greatest divergence in association results is observed between the Match and Random designs. This divergence suggests that case-control matching more effectively controls for confounding, potentially yielding more reliable SNP-level associations compared to random selection. However, this improvement in internal validity comes at a cost. Matching reduces the effective sample size by discarding unmatched controls, but it does not eliminate bias, which may lead to a loss of statistical power compared to using the full set of available controls.

This study has several limitations. First, prostate cancer cases were defined using ICD-10 codes in the UK Biobank, which lack histopathological detail and staging information. This may lead to phenotype misclassification and reduce the precision of genetic association estimates. Second, analyses were limited to individuals of European ancestry, which improves internal validity but restricts the generalisability of findings to other populations. Third, the study focused exclusively on prostate cancer as a representative phenotype. Although this enabled detailed comparison of control selection strategies, future studies should include a wider range of traits, particularly rare diseases and phenotypes with different genetic architectures.

### Conclusion

From a practical perspective, we recommend case-control matching GWAS as an efficient strategy for rapid association testing across multiple phenotypes. This approach is particularly useful when the goal is to verify known loci or replicate associations in costly sequencing contexts, rather than to discover novel variants. Looking forward, we plan to extend these evaluations to WGS and WES data and to perform external validation in deeply phenotype cohorts such as the All of Us Research Programme. This will allow us to assess generalisability across diverse ancestries and health systems.

**Key Points**

- We evaluated three strategies for selecting control groups in genome-wide association studies (GWAS): including all controls, matched controls, and random controls. All strategies demonstrated comparable capabilities in identifying single nucleotide polymorphisms (SNPs) significantly associated with prostate cancer.
- Locus analysis indicated that compared to the all controls, the matched controls, and random controls may lose up to 30% of the total loci. Using matched controls was more accurate than using random controls in identifying leading SNPs.
- Matched controls serves as a superior alternative to random controls for conducting GWAS on large population-based datasets based on cost-benefit considerations.

## Supplementary Material

Supplementary_Figures_bbag102

Supplementary_Tables_(2)_bbag102

## Availability and implementation

R code for implementing matching and random control selection is provided and available on GitHub (https://github.com/Jingzhan-Lu/GWAS-Control-Selection). All individual-level data used in this paper were obtained from the UK Biobank resource, and can be obtained from the UK Biobank at https://www.ukbiobank.ac.uk/enable-your-research/apply-for-access.
