# Supplementary material for: Impact of control selection strategies on GWAS results: a study of prostate cancer in the UK Biobank
Source: Brief Bioinform. 2026 Mar 9;27(2):bbag102. doi: 10.1093/bib/bbag102 (PMC12971001; doi:10.1093/bib/bbag102)
Supplement: Supplementary_Figures_bbag102 [file supplementary_figures_bbag102.pdf]

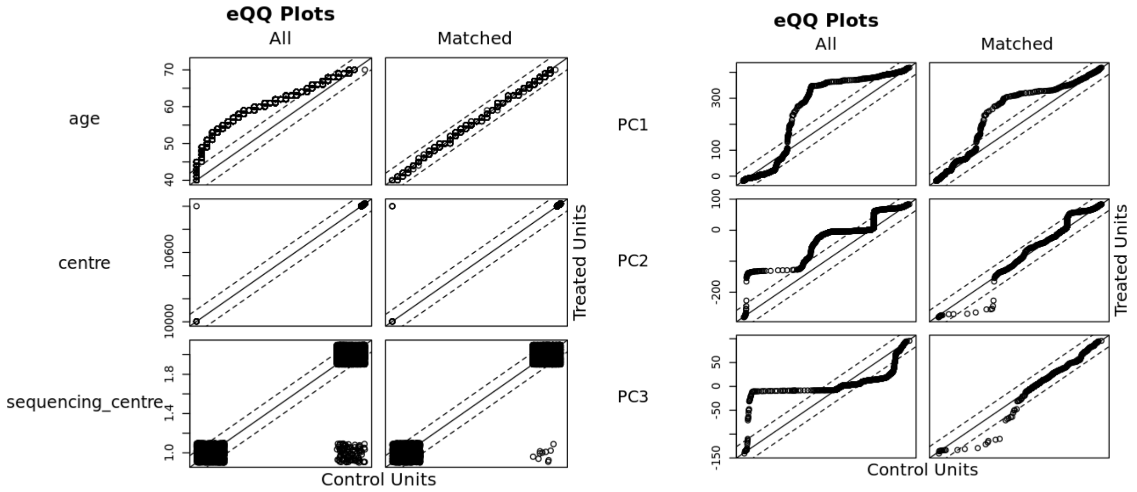

Supplementary Figure 1. Covariates balance assessment using eQQ Plots Before and After Matching

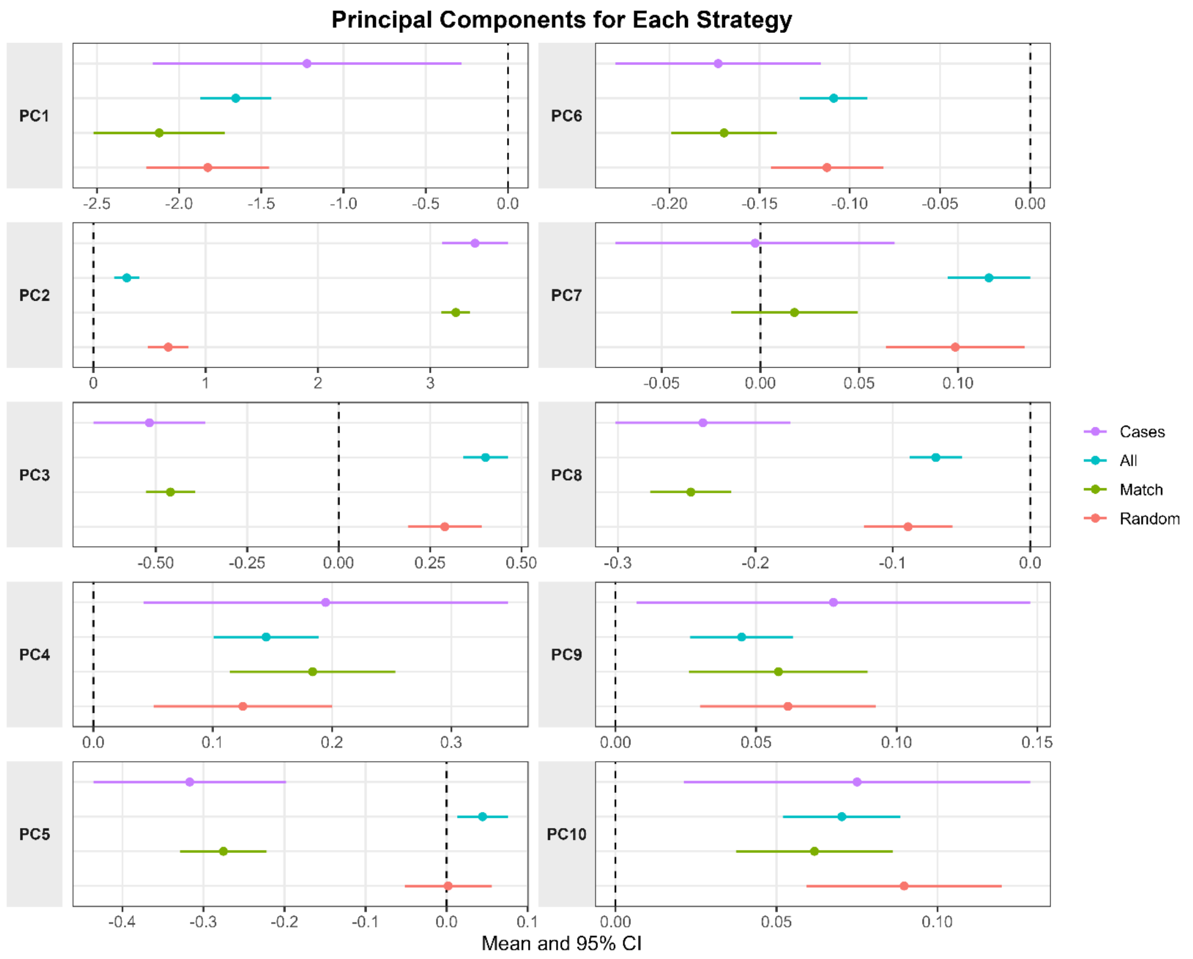

Supplementary Figure 2. Comparison of the Top 10 Principal Genetic Components for each strategy

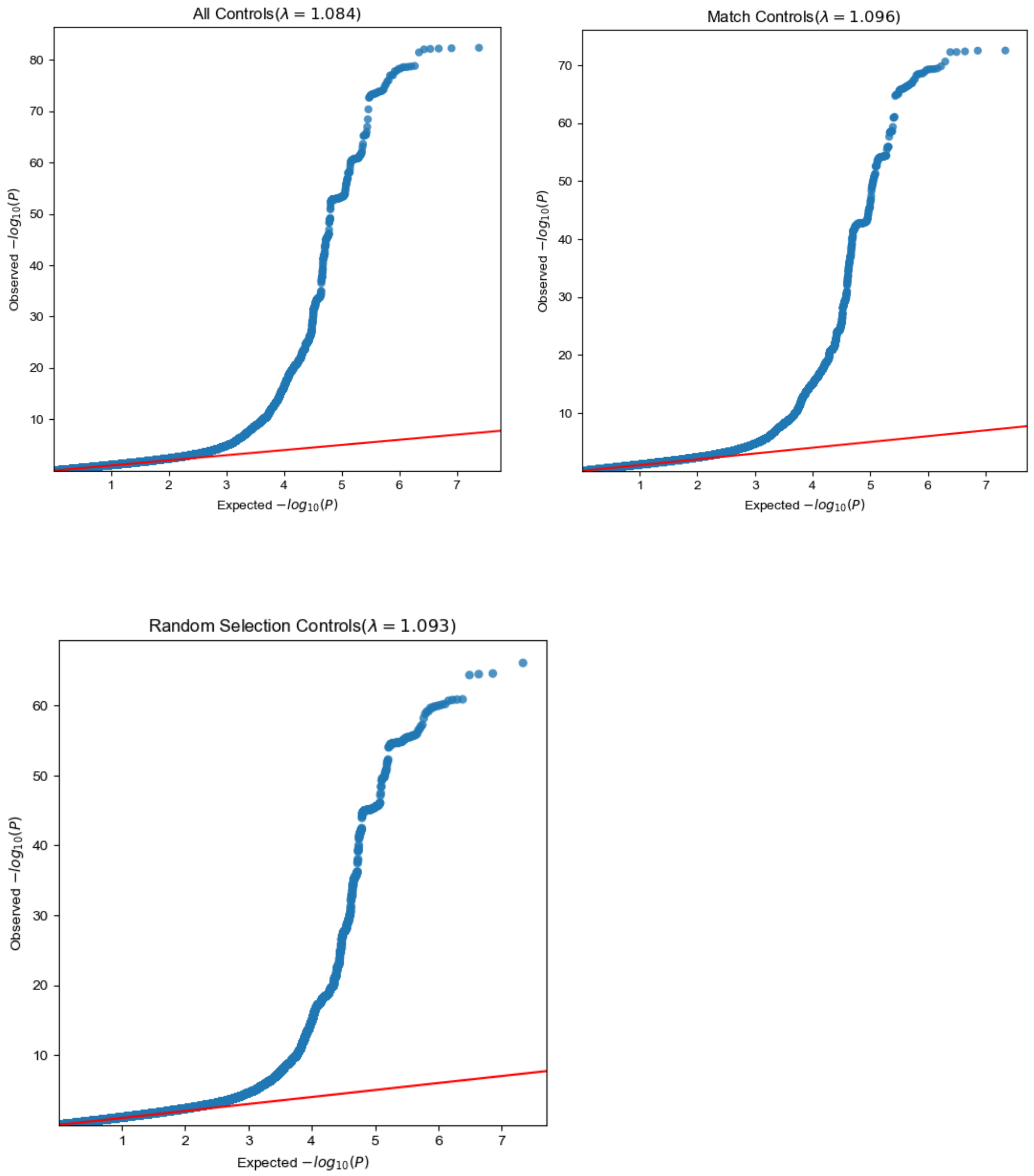

Supplementary Figure 3. QQ plots and genomic inflation factors (λ) for different control selection strategies.
